# Surrogates of Long-Term Vitamin D Exposure and Ovarian Cancer Risk in Two Prospective Cohort Studies

**DOI:** 10.3390/cancers5041577

**Published:** 2013-11-22

**Authors:** Jennifer Prescott, Kimberly A. Bertrand, Elizabeth M. Poole, Bernard A. Rosner, Shelley S. Tworoger

**Affiliations:** Channing Division of Network Medicine, Department of Medicine, Brigham and Women’s Hospital and Harvard Medical School, 181 Longwood Ave. Boston, MA 02115, USA; E-Mails: kimberly.bertrand@channing.harvard.edu (K.A.B.); nhlip@channing.harvard.edu (E.M.P.); stbar@channing.harvard.edu (B.A.R.); nhsst@channing.harvard.edu (S.S.T.)

**Keywords:** ovarian neoplasms, tumor heterogeneity, vitamin D

## Abstract

Experimental evidence and ecologic studies suggest a protective role of vitamin D in ovarian carcinogenesis. However, epidemiologic studies using individual level data have been inconsistent. We evaluated ultraviolet (UV)-B radiation, vitamin D intake, and predicted plasma 25-hydroxyvitamin D [25(OH)D] levels as long-term surrogates of vitamin D exposure within the Nurses’ Health Study (NHS) and NHSII. We estimated incidence rate ratios (RRs) and 95% confidence intervals (CIs) for risk of overall ovarian cancer and by histologic subtype using Cox proportional hazards models. Between 1976 and 2010 in NHS and 1989 and 2011 in NHSII, we identified a total of 1,225 incident epithelial ovarian cancer cases (NHS: 970, NHSII: 255) over 4,628,648 person-years of follow-up. Cumulative average UV-B exposure was not associated with ovarian cancer risk in NHS (P_trend_ = 0.08), but was associated with reduced risk in NHSII (highest *vs*. lowest category RR = 0.67; 95% CI: 0.50, 0.89; P_trend_ < 0.01). When stratified by histologic subtype, UV-B flux was positively associated with risk of serous tumors in NHS (P_trend_ < 0.01), but inversely associated in NHSII (P_trend_ = 0.01). Adjusted for confounders, ovarian cancer risk was not associated with vitamin D intake from food or supplements or with predicted 25(OH)D levels. Our study does not strongly support a protective role for vitamin D in ovarian cancer risk.

## 1. Introduction

Ovarian cancer, a relatively rare but lethal disease, is the fifth leading cause of cancer death among U.S. women [1]. Several factors that influence ovarian cancer risk have been established (e.g., parity, oral contraceptive use, family history of ovarian cancer), but most are not easily modifiable [2], making prevention a challenge. Current etiologic hypotheses suggest that ovarian cancer risk may increase with greater number of lifetime ovulations. Ovulation-induced trauma to the ovarian surface epithelium generates a local inflammatory response and stimulates proliferation, leading to an accumulation of genetic errors that may augment ovarian cancer risk [3]. Thus, identifying modifiable factors that mitigate genotoxic stress to the ovarian surface epithelium may provide alternate prevention strategies. 

Vitamin D, best known for its role in normal bone development [4,5], has nonclassical roles in immune response, differentiation, and anti-proliferative capability [6]. The vitamin D receptor, which binds to the biologically active form of vitamin D (1,25-dihydroxyvitamin D [1,25(OH)D]), is expressed within normal and malignant ovarian surface epithelium [7,8,9]. Experimental studies have demonstrated that 1,25(OH)D promotes wound healing through recruitment of immune cells, and may limit tissue damage by suppressing pro-inflammatory cytokines [10]. Furthermore, treatment of human ovarian cancer cell lines with 1,25(OH)D inhibited cell proliferation [7,11,12,13] and induced apoptosis [13].

Although several ecological studies suggest that women who live in more northern latitudes have higher rates of ovarian cancer incidence and/or mortality [14,15,16,17,18], the overall epidemiologic evidence linking the vitamin D pathway to ovarian cancer has been inconsistent. A recent systematic review of the literature concluded that individual-level epidemiologic studies have not been successful in clarifying the relationship between vitamin D exposure and ovarian cancer risk in part due to variability in study design, exposure assessment or range, and outcome definition [19]. Etiologic heterogeneity by ovarian cancer histologic subtypes [20] or tumor invasiveness [21] also may contribute to the inconsistent results, or the association between vitamin D exposures and ovarian cancer risk may be limited to subgroups of the population [20,22]. 

Given the need for additional rigorous epidemiologic studies, we assessed the risk of overall ovarian cancer and ovarian cancer histologic subtypes using multiple surrogates of long-term vitamin D exposure in the prospective Nurses’ Health Study (NHS) cohort, in which most women who developed ovarian cancer over follow-up were postmenopausal at diagnosis, and the prospective NHSII cohort, where the majority of ovarian cancer cases were premenopausal at diagnosis.

## 2. Results

Characteristics of women according to category of UV-B flux at the midpoint of follow-up (1998/1999) are shown in Table 1. Known differences between the two cohorts are the older age, larger percentage of postmenopausal women, and lower proportion of nulliparity and oral contraceptive (OC) use among women in NHS compared to NHSII. Although participants of both cohorts were initially recruited from U.S. states that covered the same range of UV-B flux exposure [100 to 180 Robertson-Berger (R-B) units], a higher proportion of NHSII participants (47%) lived in areas of high UV-B flux (>113 R-B units) compared to NHS (30%). Among NHSII women, those residing in areas of higher estimated UV-B flux were more likely to have ever used OCs and for longer duration. Among parous NHS women, those living in areas of higher UV-B flux tended to have fewer children. Postmenopausal women in both cohorts who lived in areas of higher UV-B flux were more likely to have ever used postmenopausal hormones. Age, body mass index (BMI), and dietary sources of vitamin D were similar across areas with different UV-B flux levels within each cohort.

Over the course of 2,646,148 person-years of follow-up, 970 NHS women developed incident ovarian cancer. In NHSII, 225 women were diagnosed over 1,982,500 person-years of follow-up. With the exception of average annual UV-B flux exposure at age 30, UV-B flux exposure was not significantly associated with ovarian cancer risk among NHS women (P_trend_ ≥ 0.08; Table 2) after adjusting for known ovarian cancer risk factors. Compared to NHS women in the lowest category of exposure, incidence rate ratios (RR) for the highest category of UV-B flux ranged from 1.13 [95% confidence interval (CI): 0.96, 1.33] for UV-B exposure at the time of the baseline questionnaire to 1.35 (95% CI: 1.07, 1.71; P_trend_ < 0.01) for UV-B flux at age 30 years. In a sensitivity analysis, results were similar when 1986 was used as the baseline year instead of 1976 (data not shown). In contrast, a general inverse pattern of association was observed between UV-B flux exposure at different times of life and ovarian cancer risk among NHSII participants. NHSII women in the highest category of cumulative average UV-B flux from 1989–2011 had a RR = 0.67 (95% CI: 0.50, 0.89; P_trend_ < 0.01) of developing ovarian cancer compared to women with the lowest estimated UV-B flux exposure. Heterogeneity between cohorts was apparent at virtually every time point (P_heterogeneity_ ≤ 0.06). 

An important difference between the cohorts is the higher proportion of postmenopausal women in NHS compared to NHSII (Table 1). As a result, the majority of NHS women who developed ovarian cancer were postmenopausal at diagnosis (185 premenopausal, 732 postmenopausal, 53 unknown menopausal status), whereas NHSII ovarian cancer cases were largely premenopausal (190 premenopausal, 46 postmenopausal, 19 unknown menopausal status). Upon stratifying analyses by menopausal status at diagnosis, heterogeneity in UV-B flux associations between cohorts was no longer significant (P_heterogeneity_ ≥ 0.13). Meta-analysis revealed 20% to 40% decreased risk of ovarian cancer among premenopausal women living in areas of high UV-B flux (>113 R-B units) compared to those residing in areas of low UV-B flux (<113 R-B units) at various times of life (Table 3). In contrast, exposure to high UV-B flux at these time points either was not associated or was associated with 20% to 40% increased risk of postmenopausal ovarian cancer (P_interaction_ ≤ 0.06). Heterogeneity by menopausal status was not observed in either cohort individually (P_interaction_ ≥ 0.11), although power was limited. Additionally, we did not observe heterogeneity in the association between cumulative average UV-B flux and ovarian cancer risk by age, BMI, or OC use within NHS (P_interaction_ ≥ 0.12) or NHSII (P_interaction_ ≥ 0.14).

**Table 1:** Age-standardized characteristics of NHS and NHSII participants by category of UV-B flux at the midpoint of follow-up.

Characteristics	NHS in 1998				NHSII in 1999	
	Categories of UV-B flux (R-B × 10^−4^)					
	Low (<113) (n = 27,408)	Medium (113) (n = 25,197)	High (>113) (n = 23,008)	Low (<113) (n = 31,679)	Medium (113) (n = 22,903)	High (>113) (n = 48,322)
Age, years ^a^	61.6 (7.1)	61.6 (7.1)	63.0 (7.2)	42.2 (4.7)	42.7 (4.5)	42.4 (4.6)
Cumulative average UV-B flux, R-B × 10^−4^	105 (3)	113 (0)	149 (22)	104 (3)	113 (0)	145 (21)
UV-B flux at birth, R-B × 10^−4^	107 (9)	114 (7)	126 (25)	107 (11)	114 (9)	132 (24)
UV-B flux at age 15, R-B × 10^−4^	107 (8)	113 (6)	128 (26)	106 (9)	113 (6)	135 (24)
UV-B flux at age 30, R-B × 10^−4^	106 (8)	113 (6)	139 (28)	105 (10)	114 (7)	143 (23)
UV-B flux at baseline, R-B × 10^−4^	105 (4)	113 (0)	143 (28)	104 (4)	113 (0)	145 (23)
BMI, kg/m^2^	26.4 (5.2)	26.8 (5.4)	25.9 (5.2)	26.0 (6.0)	26.4 (6.1)	25.9 (5.9)
Cumulative average total dietary vitamin D ^b^, IU/day	351 (203)	339 (201)	359 (224)	388 (222)	364 (217)	369 (224)
Cumulative average energy-adjusted food vitamin D ^b^, IU/day	213 (87)	199 (82)	203 (87)	253 (110)	234 (105)	236 (110)
Cumulative average supplemental vitamin D ^b^, IU/day	125 (163)	128 (164)	148 (192)	133 (168)	128 (164)	135 (171)
Age at menarche, years	12.5 (1.4)	12.6 (1.4)	12.6 (1.4)	12.4 (1.4)	12.4 (1.4)	12.4 (1.4)
Ever use of oral contraceptives, %	49	46	56	82	83	89
Duration of OC use, years ^c^	4.1 (3.8)	4.0 (3.7)	4.4 (4.0)	4.7 (4.2)	5.0 (4.4)	5.6 (4.7)
Ever parous, %	95	95	93	81	82	78
Number of children among parous women	3.4 (1.6)	3.2 (1.5)	3.0 (1.4)	2.3 (1.0)	2.3 (1.0)	2.2 (0.9)
Tubal ligation, %	19	22	19	22	28	25
Family history of ovarian cancer, %	3	3	3	2	2	2
Postmenopausal, %	86	85	85	4	5	4
Ever use of postmenopausal hormones ^d^, %	50	52	67	68	71	76

Values are means (SD) or percentages and are standardized to the age distribution of the study population. UV-B = ultraviolet B; R-B × 10^−4^ = Robertson-Berger units; BMI = body mass index; IU = international units; OC = oral contraceptives; ^a^ Value is not age adjusted; ^b^ Starting with baseline dietary questionnaire (1980 in NHS, 1991 in NHSII); ^c^ Among ever OC users; ^d^ Among postmenopausal women.

**Table 2:** Associations between surrogates of vitamin D exposure and epithelial ovarian cancer risk in the NHS and NHS II.

Exposure	Nurses’ Health Study				Nurses’ Health Study II						
	Cases, N	Person-years	Multivariable-adjusted RR ^a^	95% CI	P-trend ^b^	Cases, N	Person-years	Multivariable-adjusted RR ^a^	95% CI	P-trend ^b^	P-het ^c^
UV-B flux at birth, R-B × 10^−4^											
Low (<113)	166	329,536	1.00	ref	0.11	83	535,110	1.00	ref	0.14	0.06
Medium (113)	158	314,502	0.95	0.76–1.18		55	413,614	0.88	0.62–1.24		
High (>113)	97	142,714	1.21	0.93–1.56		68	587,015	0.78	0.56–1.07		
UV-B flux at age 15, R-B × 10^−4^											
Low	168	330,546	1.00	ref	0.12	73	412,386	1.00	ref	0.06	0.02
Medium	167	323,148	0.98	0.79–1.21		49	326,486	0.86	0.59–1.23		
High	95	140,306	1.20	0.93–1.56		56	460,142	0.71	0.50–1.00		
UV-B flux at age 30, R-B × 10^−4^											
Low	160	325,939	1.00	ref	<0.01	69	370,470	1.00	ref	0.05	<0.01
Medium	151	303,456	0.98	0.79–1.23		42	291,105	0.78	0.53–1.15		
High	132	178,182	1.35	1.07–1.71		66	515,274	0.69	0.49–0.96		
UV-B flux at baseline, R-B × 10^−4^											
Low	351	1,040,302	1.00	ref	0.15	96	629,292	1.00	ref	0.02	0.01
Medium	366	1,020,194	1.04	0.90–1.21		63	476,575	0.86	0.62–1.18		
High	253	585,652	1.13	0.96–1.33		96	876,633	0.70	0.53–0.93		
UV-B flux cumulative updated average from baseline questionnaire, R-B × 10^−4^											
Low	326	986,280	1.00	ref	0.08	95	613,895	1.00	ref	<0.01	<0.01
Medium	317	927,286	1.02	0.87–1.19		60	444,825	0.87	0.63–1.21		
High	327	732,582	1.14	0.97–1.33		100	923,780	0.67	0.50–0.89		
Cumulative average vitamin D intake from food, IU/day ^d^											
<200	406	952,357	1.00	ref	0.61	77	597,819	1.00	ref	0.74	0.36
200–299	234	533,531	0.96	0.81–1.13		78	480,302	1.26	0.92–1.72		
300+	91	217,913	0.96	0.76–1.20		45	352,606	1.03	0.71–1.50		
Cumulative average supplemental vitamin D intake, IU/day											
None	266	751,728	1.00	ref	0.71	58	539,404	1.00	ref	0.43	0.25
<200	238	463,540	1.15	0.95–1.39		83	396,170	1.78	1.24–2.56		
200+	227	488,533	1.07	0.89–1.29		59	495,153	1.05	0.72–1.52		
Cumulative average predicted 25(OH)D score											
Lowest tertile	139	300,915	1.00	ref	0.07	60	357,464	1.00	ref	0.05	<0.01
Intermetdiate tertile	148	330,632	0.91	0.72–1.15		70	454,709	0.98	0.69–1.38		
Highest tertile	154	230,673	1.27	1.00–1.61		51	470,741	0.68	0.48–1.00		

^a^ Adjusted for age (as the time scale), duration of oral contraceptive use, number of pregnancies, tubal ligation, menopausal status, ever use of postmenopausal hormones, and first-degree family history of ovarian cancer; ^b^ Trend test based on median of the category; ^c^ Heterogeneity between cohorts assessed using trend variables; ^d^ Additionally adjusted for total caloric intake.

**Table 3:** Risk of ovarian cancer by menopausal status at diagnosis ^a^.

Exposure	Premenopausal					Postmenopausal					
	Cases, N	Person-years	Multivariable-adjusted RR ^b^	95% CI	P-trend ^c^	Cases, N	Person-years	Multivariable-adjusted RR ^b^	95% CI	P-trend ^c^	P-int ^d^
UV-B flux at birth, R-B × 10^−4^											
Low (<113)	70	470,702	1.00	ref	0.21	167	360,834	1.00	ref	0.59	0.06
Medium (113)	44	365,110	0.80	0.55–1.17		165	331,549	1.01	0.82–1.26		
High (>113)	53	498,500	0.77	0.53–1.11		107	195,134	1.21	0.89–1.64		
UV-B flux at age 15, R-B × 10^−4^											
Low	59	350,076	1.00	ref	0.04	170	360,316	1.00	ref	0.49	0.02
Medium	41	277,252	0.85	0.57–1.27		172	339,988	1.02	0.82–1.26		
High	38	369,923	0.66	0.43–1.00		105	193,486	1.13	0.76–1.69		
UV-B flux at age 30, R-B × 10^−4^											
Low	57	313,749	1.00	ref	0.02	160	351,564	1.00	ref	<0.01	<0.01
Medium	35	248,444	0.76	0.50–1.16		156	316,438	1.04	0.83–1.29		
High	43	409,234	0.61	0.40–0.91		146	241,551	1.37	1.09–1.73		
UV-B flux at baseline, R-B × 10^−4^											
Low	148	902,035	1.00	ref	0.03	270	661,894	1.00	ref	0.05	<0.01
Medium	126	759,919	0.98	0.77–1.25		283	626,680	1.07	0.91–1.27		
High	101	902,447	0.75	0.58–0.98		225	448,003	1.21	1.01–1.45		
UV-B flux cumulative updated average from baseline questionnaire, R-B × 10^−4^											
Low	145	884,813	1.00	ref	0.07	248	614,338	1.00	ref	0.87	<0.01
Medium	117	718,793	0.97	0.76–1.24		241	552,788	1.04	0.87–1.24		
High	113	960,795	0.78	0.59–1.03		289	569,449	1.11	0.77–1.60		
Cumulative average vitamin D intake from food, IU/day ^e^											
<200	119	748,511	1.00	ref	0.20	341	695,347	1.00	ref	0.31	0.05
200–299	96	523,242	1.27	0.91.67		206	431,725	0.94	0.79–1.12		
300+	57	358,610	1.22	0.81.69		71	180,409	0.66	0.27–1.62		
Cumulative average supplemental vitamin D intake, IU/day											
None	128	741,436	1.00	ref	0.18	181	459,440	1.00	ref	0.91	0.19
<200	74	379,762	1.13 ^f^	0.51–2.50		233	431,678	1.25	1.02–1.54		
200+	70	509,165	0.87	0.64–1.18		204	416,361	1.15	0.93–1.41		
Cumulative average predicted 25(OH)D score											
Lowest tertile	65	360,441	1.00	ref	0.99 ^f^	134	297,939	1.00	ref	0.72	0.18
Intermediate tertile	68	457,829	0.90	0.64–1.27		150	327,512	1.07	0.61–1.87		
Highest tertile	58	443,145	1.00^f^	0.43–2.35		147	258,269	1.05	0.61–1.80		

^a^ DerSimonian-Laird estimators for random effects models were used to combine cohort results; ^b^ Adjusted for age (as the time scale), duration of oral contraceptive use, number of pregnancies, tubal ligation, ever use of postmenopausal hormones (among postmenopausal women), and first-degree family history of ovarian cancer; ^c^ Trend test based on median of the category; ^d^ Interaction by menopausal status assessed using trend variables; ^e^ Additionally adjusted for total caloric intake; ^f^ Significant heterogeneity in estimates between cohort.

Rates of mucinous, endometrioid, and clear cell tumors peak at earlier ages than serous tumors. Additionally, younger women are more likely to be diagnosed with early stage disease and have a more favorable prognosis than older women [2]. Therefore, we examined whether ovarian cancer risk associated with UV-B flux differed by histologic subtype (serous, mucinous, endometrioid/clear cell), tumor invasiveness (invasive *vs*. borderline) or aggressiveness (death from invasive ovarian cancer within 3 years of diagnosis *vs*. less aggressive invasive disease; Table 4). Similar to the overall results, significant heterogeneity was observed between cohorts among women who developed serous tumors (P_heterogeneity_ < 0.01). NHS women residing in areas of high *versus* low UV-B flux (>113 *versus* < 113 R-B units) were at increased risk of serous ovarian cancer (RR = 1.34; 95% CI: 1.10, 1.64). In contrast, within NHSII, the comparable RR for serous ovarian tumors was 0.59 (95% CI: 0.39, 0.89). Residing in areas of higher UV-B flux was not associated with mucinous or endometrioid/clear cell tumors in either cohort (P_trend_ ≥ 0.14), although case numbers were low. Whereas UV-B flux was not associated with borderline tumors, a similar heterogeneous pattern between cohorts was observed for invasive disease (P_heterogeneity_ < 0.01), which may be driven by the large proportion of serous tumors within this group (65.8% in NHS, 44.7% in NHSII). UV-B flux was not associated with risk of developing an aggressive invasive tumor, but was associated with less aggressive invasive tumors, again with opposing directions of association between the cohorts (P_heterogeneity_ < 0.01). Considering the lack of a biological rationale for etiologic differences in serous tumor development or tumor aggressiveness by menopausal status, we explored additional known dissimilarities between the cohorts [23] as potential confounders. Results were similar after adjusting for duration of estrogen-only postmenopausal hormone use, duration of breastfeeding, physical activity, or smoking status.

Ovarian cancer risk was not associated with dietary vitamin D intake from food or supplements in either cohort (Table 2). We observed some evidence of heterogeneity for food vitamin D intake with ovarian cancer risk by menopausal status (P_interaction_ = 0.05; Table 3). However, in stratified analysis, food vitamin D was not significantly associated with risk of either pre- or postmenopausal ovarian cancer. With the exception of a reduced risk of mucinous tumors observed among NHS women who consumed ≥200 IU of supplemental vitamin D per day *versus* none (RR = 0.47; 95% CI: 0.23, 0.99; P_trend_ = 0.05), dietary sources of vitamin D intake were not associated with specific histologic subtypes, invasiveness, or aggressiveness of ovarian tumors in either cohort (data not shown). Tests for interaction suggested potential differences in ovarian cancer risk associated with vitamin D intake from food when stratified by ever use of OCs in NHS (P_interaction_ = 0.04) and by menopausal status (P_interaction_ = 0.03) or BMI (P_interaction_ = 0.03) in NHSII. However, no clear patterns of association emerged within strata and are likely due to chance. Risk of ovarian cancer with supplemental vitamin D intake did not differ by age, menopausal status, BMI, or ever use of OCs in either cohort (P_interaction_ ≥ 0.11).

**Table 4:** Cumulative average UV-B flux association with histologic subtypes of ovarian cancer in NHS and NHS II.

Cumulative average UV-B flux, R-B × 10^−4^ Person-years	Nurses’ Health Study				Nurses’ Health Study II				P-het ^c^
	Low	Medium	High	P-trend ^b^	Low	Medium	High	P-trend ^b^	
	986,280	927,286	732,582		613,895	444,825	923,780		
Serous, N	183	205	213		46	30	42		
Multivariate-adjusted RR ^a^	1.00	1.19	1.34	<0.01	1.00	0.90	0.59	0.01	<0.01
(95% CI)	(ref)	(0.97–1.45)	(1.10–1.64)		(ref)	(0.57–1.43)	(0.39–0.89)		
Mucinous, N	33	25	17		8	6	12		
Multivariate-adjusted RR ^a^	1.00	0.79	0.62	0.14	1.00	1.04	0.95	0.87	0.47
(95% CI)	(ref)	(0.47–1.32)	(0.34–1.13)		(ref)	(0.36–3.01)	(0.39–2.32)		
Endometrioid/clear cell, N	58	53	54		25	15	33		
Multivariate-adjusted RR ^a^	1.00	0.92	1.04	0.72	1.00	0.85	0.82	0.52	0.47
(95% CI)	(ref)	(0.63–1.34)	(0.71–1.51)		(ref)	(0.45–1.61)	(0.49–1.38)		
Invasive tumors, N	272	274	282		77	44	78		
Multivariate-adjusted RR ^a^	1.00	1.06	1.17	0.06	1.00	0.79	0.65	0.01	<0.01
(95% CI)	(ref)	(0.90–1.25)	(0.99–1.39)		(ref)	(0.55–1.15)	(0.47–0.89)		
Borderline tumors, N	37	30	25		16	14	16		
Multivariate-adjusted RR ^a^	1.00	0.83	0.86	0.66	1.00	1.26	0.65	0.11	0.33
(95% CI)	(ref)	(0.51–1.35)	(0.51–1.44)		(ref)	(0.61–2.58)	(0.32–1.30)		
Rapidly fatal disease ^d^, N	133	135	120		16	7	18		
Multivariate-adjusted RR ^a^	1.00	1.07	1.02	0.97	1.00	0.60	0.70	0.47	0.50
(95% CI)	(ref)	(0.84–1.36)	(0.79–1.31)		(ref)	(0.25–1.47)	(0.36–1.38)		
Less aggressive disease ^d^, N	130	130	142		56	31	49		
Multivariate-adjusted RR ^a^	1.00	1.04	1.26	0.04	1.00	0.76	0.55	<0.01	<0.01
(95% CI)	(ref)	(0.82–1.33)	(0.99–1.61)		(ref)	(0.49–1.18)	(0.38–0.81)		

^a^ Adjusted for age (as the time scale), duration of oral contraceptive use, number of pregnancies, tubal ligation, menopausal status, ever use of postmenopausal hormones, and first-degree family history of ovarian cancer; ^b^ Trend test based on median of the category; ^c^ Heterogeneity between cohorts assessed using trend variables; ^d^ Death from invasive ovarian cancer within 3 years of diagnosis was considered rapidly fatal. Women who did not die within 3 years from invasive ovarian cancer were considered to have less aggressive disease.

Predicted 25-dihydroxyvitamin D [25(OH)D] levels were marginally positively associated with ovarian cancer risk in the NHS. Participants in the highest predicted tertile of predicted 25(OH)D had a RR = 1.27 (95% CI: 1.00, 1.61; P_trend_ = 0.07) compared to those in the lowest tertile. In contrast, NHSII women in the highest tertile of predicted 25(OH)D levels were at reduced risk of ovarian cancer (RR = 0.68; 95% CI: 0.48, 1.00; P_trend_ = 0.05) compared to women in the lowest tertile (Table 2). Given that BMI is one of the strongest predictors of circulating 25(OH)D levels (Table A1) and has been suggestively associated with ovarian cancer risk in premenopausal women [24], we further adjusted for BMI as a potential confounder. Adding BMI to the model completely attenuated the association between the predicted 25(OH)D score and ovarian cancer risk in NHSII (highest *vs.* lowest tertile RR = 1.18; 95% CI: 0.71, 1.94; P_trend_ = 0.61). In NHS, the association was slightly attenuated when BMI was included in the model. Given concerns about over-controlling for BMI, we conducted a sensitivity analysis where we removed the BMI component when calculating the predicted 25(OH)D score. The modified score was not associated with ovarian cancer risk in either cohort regardless of whether BMI was included in the model (P_trend_ ≥ 0.39). Predicted 25(OH)D levels were not associated with ovarian cancer risk when stratified by menopausal status (Table 3). We also did not observe heterogeneous associations of predicted 25(OH)D levels with ovarian cancer risk in analyses stratified by age, BMI, or ever use of OCs within either cohort (P_interaction_ ≥ 0.12). The predicted 25(OH)D score was not associated with risk of ovarian cancer histologic subtypes or tumor invasiveness in either cohort (data not shown). Predicted 25(OH)D levels were not associated with development of rapidly fatal ovarian tumors in NHS, but women in the highest *versus* lowest tertile of predicted 25(OH)D levels were at increased risk of less aggressive invasive tumors (RR = 1.46; 95% CI: 1.01, 2.11; P_trend_ = 0.02). In NHSII, predicted 25(OH)D levels were not associated with risk of aggressive or less aggressive invasive tumors.

## 3. Discussion

In the current study, we used UV-B flux based on state of residence, dietary sources of vitamin D intake, and predicted 25(OH)D score as long-term surrogates of vitamin D exposure in two prospective cohorts. We did not observe consistent evidence linking the vitamin D pathway with reduced ovarian cancer risk. Among NHS women, these exposures were not associated with a lower risk of ovarian cancer, and in some instances were associated with an increased risk, particularly of serous and less aggressive invasive tumors. However, among NHSII women, living in areas of high UV-B flux generally was associated with a lower risk of ovarian cancer, including serous and less aggressive invasive tumors. The predicted 25(OH)D score also was associated with a lower risk in NHSII, although this association was attenuated when adjusted for BMI. Interestingly, BMI did not confound the association with UV-B flux. 

Divergent UV-B flux associations were observed for ovarian cancer between the two cohorts. We hypothesized that the observed associations with overall ovarian cancer differed because younger women tend to develop tumors with better prognosis than older women. Similar opposing relationships were observed when stratified by menopausal status, in which UV-B flux appeared associated with reduced premenopausal ovarian cancer risk, but a possibly increased postmenopausal ovarian cancer risk depending on timing of exposure. Interaction by menopausal status (P_interaction_ ≥ 0.11) was not observed in either cohort individually, although sample sizes within strata were small. Stratifying by subtypes of ovarian cancer revealed that discrepant associations between cohorts were particularly evident for risk of serous and less aggressive invasive tumors. While menopausal status may account for the discrepant results between cohorts, we cannot exclude the possibility of a cohort effect. By design, the oldest woman in NHSII is still younger than the youngest woman in NHS for any given year (*i.e.*, no overlap in year of birth). Known differences between the cohorts with respect to duration of estrogen-only postmenopausal hormone use, duration of breastfeeding, total physical activity, or smoking status [23] did not account for the opposing relationships. The inconsistent results between the cohorts could potentially be due to temporal and geographic differences of an unmeasured confounder.

A shift in reproductive behaviors is apparent among participants of the two cohorts, reflecting general population trends in the U.S. Oral contraceptives first became commercially widely available in 1960 and were quickly adopted by married women to regulate childbearing [25]. Most women born before 1945, a birth cohort to which ~94% of NHS participants belong, began using oral contraceptives after their first full term pregnancy. As social norms changed and younger and unmarried women gained increased access to oral contraceptives, age at first use declined with a concomitant increase in use prior to first pregnancy [26]. Some studies have suggested that age at first oral contraceptive use and/or formulation may influence ovarian cancer risk [27,28,29,30,31]. Therefore, regional differences in adopting oral contraceptive use, prescribed formulation type, and/or potency over time may account for the inconsistent UV-B flux associations observed between the two cohorts, although the proportional hazards assumption was not violated in either cohort (P ≥ 0.19). While we did not observe significant interactions by ever use or duration of OCs (P_interaction_ ≥ 0.12), evidence of a positive association between UV-B flux and overall ovarian cancer risk was observed among NHS women who had ever used oral contraceptives (P_trend_ = 0.05), but not among never users (P_trend_ = 0.93).

In general, the epidemiologic literature does not support a strong association between vitamin D exposure and ovarian cancer risk [19]. The hypothesis is supported mainly by ecological studies comparing latitude, regional solar irradiance, or skin cancer rates to ovarian cancer incidence and/or mortality rates [14,15,16,18]. UV-B radiation remained significantly associated with ovarian cancer incidence rates after adjusting for fertility rates at ages 15 to 19 in an ecological study of 175 countries [15]. However, fertility does not strongly correlate with oral contraceptive use [25], the rates of which vary by country [32]. Furthermore, not all ecological studies observed an association between residential UV irradiance or non-melanoma skin cancer rates with ovarian cancer incidence rates [17,33,34,35]. While ecological studies are useful in generating hypotheses, they are extremely sensitive to confounders and do not necessarily reflect associations on the individual level [35]. 

A population-based case-control study in Australia observed reduced risk of ovarian cancer among women in the highest tertile of lifetime ambient UV radiation (multivariate-adjusted OR = 0.73; 95% CI: 0.57, 0.95). The association appeared stronger for women diagnosed with borderline tumors (OR = 0.51; 95% CI: 0.32, 0.81) and displayed similar ~25% to 30% decreased risks for invasive serous, mucinous, and endometrioid tumors [21]. While we observed a reduced risk of serous tumors in the younger NHSII cohort for women living in areas of higher UV-B flux, UV-B flux was not associated with mucinous or endometrioid/clear cell tumors. A 35% decreased risk of both invasive and borderline tumors was observed among NHSII women in areas of higher compared to lower UV-B flux. In contrast, increased risk of serous or invasive tumors was observed among women in the NHS residing in areas of higher UV-B flux. Similar patterns of association were observed for risk of less aggressive invasive tumors. The Australian study used NASA’s Total Ozone Mapping Spectrometer (TOMS) satellite data, which correlates well with UV-B flux estimates on clear days [36]. UV radiation exposure based on TOMS satellite data, also was used in a population-based study of 1,334 case and 1,679 control women from Western Washington state [37] and the prospective National Institutes of Health-AARP Diet and Health Study [38]. Overall ovarian cancer risk was not associated with UV radiation in these studies. The Western Washington state case-control study reported a weak increased risk of serous tumors among women with increasing estimated UV radiation exposure in the 10 years prior to diagnosis, but not for lifetime exposure [37]. Overall, women in the Australian study resided in areas of higher UV-B flux than women in the U.S. studies. Thus, differences in the range of solar irradiation exposure may account for some of the discrepancy in results. 

Vitamin D intake from food or supplements was not associated with overall ovarian cancer risk in either NHS or NHSII. Our results are consistent with the majority of studies assessing dietary intake of vitamin D from food and/or supplements as a risk factor for ovarian cancer. Two population-based case-control [20,39] and two prospective [40,41] studies generally did not observe an association of total, food, and/or supplemental vitamin D with ovarian cancer risk overall or by histology, although two hospital-based studies reported a lower risk [42,43]. A pooled analysis of 2,132 ovarian cancer cases among 553,217 women from 12 prospective cohorts, including NHS and NHSII participants, observed a positive association between vitamin D intake from food and overall ovarian cancer risk among women who consumed ≥400 IU/day compared to women with <100 IU/day (P_trend_ = 0.04) [44]. The lack of consistency suggests dietary sources of vitamin D intake are not related to ovarian cancer. Given that the hypothesized protective effect involves the vitamin D pathway, the lack of an association is not surprising given that ≥ 90% of circulating 25(OH)D is synthesized cutaneously in humans from UV radiation exposure [45]. Thus, vitamin D intake contributes to a relatively small proportion of vitamin D exposure in most populations.

Similar inconsistencies in ovarian cancer risk have been observed in studies that measured circulating levels of 25(OH)D, which accounts for multiple determinants of overall vitamin D status (e.g., sun exposure, skin pigmentation, dietary intake) [46,47,48]. In two independent samples of women from the Finnish Maternity Cohort, ~3-fold increased risk of overall ovarian cancer was observed among women (who were necessarily premenopausal) who were insufficient for 25(OH)D levels (<75 nmol/L) compared to women who were sufficient [49,50]. In contrast, no association between circulating 25(OH)D and ovarian cancer risk was observed in a pooled analysis of mostly postmenopausal women from case-control studies nested within eight prospective cohorts, including the NHS, with the exception of a suggested reduced risk among women who were overweight or obese [22]. Together, the studies suggest that reduced risk of ovarian cancer associated with higher levels of circulating 25(OH)D may be limited to premenopausal women, which coincides with the reduced risk of ovarian cancer associated with UV-B flux among NHSII participants or when analyses were stratified by menopausal status. A reduced risk of ovarian cancer also was observed among NHSII participants with higher levels of predicted 25(OH)D levels. However, the association was attenuated after adjustment for BMI.

The use of two large prospective cohorts with detailed information on ovarian cancer risk factors collected and updated over 34 years of follow-up are major strengths of our study. The wealth of data available allowed us to examine ovarian cancer risk associated with estimates of ambient UV exposure at various times of life as well as other surrogates for vitamin D exposure. We derived estimates of UV-B exposure based on the Robertson-Berger meter network, which is calibrated to provide measurements of solar UV-B irradiation that correspond with the human erythemal (sunburn) response [51] as an objective measure of ambient UV radiation exposure. 

Our study has several limitations that must be acknowledged. Participants within our cohorts are female registered nurses recruited from a small proportion of U.S. states. The target population was chosen to maximize internal validity through enhanced reporting accuracy of exposures and health outcomes and a high rate of follow-up, with the trade-off that the women would not be representative of the general U.S. population. Even so, as underlying biology is likely very similar across ethnic groups and social class, observations made in our cohorts may apply more broadly. Further, the states from which participants were recruited represent a large proportion (100 to 180 R-B units) of the average annual UV-B irradiation in the U.S. As participants moved over the course of follow-up, the distribution of average annual UV-B flux estimates expanded to cover the entire range of average annual UV-B flux (93 to 196 R-B units). The use of residence-based UV-B flux is limited by the fact that individual level exposure to solar irradiation may vary depending on actual time spent outdoors and sun-protective behaviors of participants, resulting in exposure misclassification. However, given the prospective study design and the 2-year time lag between exposure assessment and ovarian cancer diagnosis we incorporated into our analysis (see Experimental Section), any misclassification would have been non-differential with respect to disease, attenuating effect estimates. Although a single assessment may not fully reflect lifetime sun protective behaviors, our results were similar after we adjusted analyses for time spent outdoors and sunscreen use, which were asked on the 1980 questionnaire in NHS and on the 1993 questionnaire in NHSII. Another limitation to consider is that while we adjusted for established ovarian cancer risk factors, confounding by potential unmeasured risk factors such as timing, formulation and/or potency of oral contraceptives, or unidentified environmental exposures cannot be ruled out. The positive association between UV-B flux and overall ovarian cancer risk observed among NHS women appeared limited to women who ever used oral contraceptives, indicating that unmeasured confounding may have influenced our results. 

## 4. Experimental

### 4.1. Study Populations

The NHS is an ongoing prospective cohort following 121,700 female registered nurses who were 30–55 years of age and from 11 U.S. states (CA, CT, FL, MA, MI, MD, NJ, NY, OH, PA, TX) in 1976. NHSII, a similar cohort begun in 1989, enrolled 116,430 female registered nurses aged 25–42 years from 14 US states (CA, CT, IA, IN, KY, MA, MI, MO, NY, NC, OH, PA, SC, TX). At baseline and biennially thereafter, participants from each cohort completed self-administered questionnaires that were used to gather and update detailed information on anthropometric and lifestyle variables, menstrual and reproductive factors, and medical history, as well as identify newly diagnosed cancers and other diseases. Blood samples were collected during 1989 and 1990 from a subset of 32,826 NHS participants and from 1996 to 1999 from 29,611 NHSII participants. Follow-up was high, obtaining >90% of total possible person-years within each cohort. Vital status was ascertained through next-of-kin, the U.S. Postal Service, and the National Death Index; both methods have identified an estimated 98% of deaths in the cohort [52].

Completion of the self-administered questionnaire was considered to imply informed consent. The NHS and NHSII protocols were approved by the Institutional Review Board at the Brigham and Women’s Hospital, Boston, MA, USA.

### 4.2. Case Ascertainment

We identified women reporting a new ovarian cancer diagnosis on each questionnaire. For all reported cases and deaths due to ovarian cancer, we either asked permission from the participant or next of kin to obtain medical records pertaining to the ovarian cancer diagnosis or retrieved information about the case from the appropriate state or federal cancer registry. A gynecologic pathologist blinded to exposure status reviewed the medical records or registry data to confirm the diagnosis, as well as abstract stage, histology, and invasiveness. In a subset of 215 ovarian cancer cases, concordance between the medical records and the pathologist’s review was 98% for invasiveness and 83% for histologic type [53]. Our analysis includes all confirmed cases of epithelial ovarian cancer and primary peritoneal cancer diagnosed between the baseline questionnaire and end of follow-up (NHS: 1 June 2010; NHSII: 1 June 2011).

### 4.3. Exposure Data

Average annual UV-B flux is a composite measure of biologically effective UV radiation at the earth’s surface based on latitude, altitude, and cloud cover [36,54]. Weighted counts per 10,000 of UV-B radiation collected by the Robertson-Berger meter network across the U.S. [51] were used to estimate UV-B flux for participants at birth, age 15 years, age 30 years, at baseline, and every 2 years since 1986 in NHS and 1989 in NHSII according to state of residence. In the NHS, if a woman lived in the same state in 1976 as in 1986 (~90% of participants), we assumed she lived in the same state for the intervening years. If state of residence in 1976 and 1986 was different, we assumed that in 1978 and 1980 she lived in the state listed in 1976, and in 1982 and 1984 she lived in the state listed in 1986. State of residence at birth, age 15, and age 30 were asked on the NHS 1992 questionnaire. In NHSII, state of residence at birth was ascertained on the 1989 questionnaire, while state of residence at ages 15 and 30 was reported on the 1993 questionnaire. Throughout follow-up, current residence was obtained using available mailing addresses of participants. Although not a direct measure of sun exposure, UV-B flux is associated with melanoma risk [55], suggesting that it is a reasonable proxy. Moreover, it is an objective exposure metric that does not rely on personal recall of time spent outdoors. 

In 1980, a 61-item semi-quantitative food frequency questionnaire (FFQ) was sent to NHS participants to obtain dietary information. Expanded FFQs were subsequently sent approximately every 4 years. Dietary information was first collected from NHSII women in 1991 and every 4 years thereafter using a similar FFQ that asked about annual average intake of over 130 individual foods and 22 beverages. All FFQs inquired about how often, on average, participants consumed each food using standard portion sizes. Nine responses were possible, ranging from never or less than once per month to 6 or more times per day. Intake of dietary vitamin D and other nutrients was calculated by multiplying the portion size of a single serving of each food by its reported frequency of intake, then multiplying the total amount consumed by the nutrient content of the food, and summing the nutrient contributions of all food items using U.S. Department of Agriculture food composition data [56], while also taking dietary supplements into account. The reproducibility of the FFQ has been reported elsewhere [57,58,59]. Vitamin D intake has been validated using plasma concentrations of 25(OH)D, a relatively stable indicator of vitamin D status [60], with a reported correlation of 0.25 (*p* < 0.01) [61,62].

Multivariable linear regression models to predict 25(OH)D levels within each cohort previously were developed and validated [63]. Based on these existing models, separate linear regression models were fit among premenopausal and postmenopausal women to create menopause status-specific predictor scores for this analysis. Plasma 25(OH)D measurements were available from 2,431 premenopausal and 3,101 postmenopausal women who served as controls in previous nested case-control studies of chronic disease. Plasma 25(OH)D levels were determined by radioimmunoassay or chemiluminescence immunoassay, as previously described [64,65,66], between 1993 and 2012. Intra-assay coefficients of variation (CV) from blinded, replicate, quality-control samples were <15% for twenty-three out of thirty laboratory batches; the highest CV was 21.6%. Mean (standard deviation) plasma 25(OH)D concentrations were 26.0 (10.2) ng/mL in premenopausal women [26.1 (10.8) ng/mL in NHS, 26.0 (10.0) ng/mL in NHSII] and 26.6 (10.6) ng/mL in postmenopausal women [26.8 (10.7) ng/mL in NHS, 24.5 (9.6) ng/mL in NHSII].

Predictors of plasma 25(OH)D levels were categorized as follows: race/ethnicity (white, black, Hispanic, Asian, and other; a proxy for skin pigmentation), BMI (<22.0, 22.0–24.9, 25.0–29.9, 30–34.9, 35+ kg/m^2^), total leisure-time physical activity (<3, 3–8.9, 9–17.9, 18–26.9, 27+ METS/week; a proxy of outdoor sunlight exposure), energy-adjusted [67] vitamin D from food (<100, 100–199, 200–299, 300–399, 400+ IU/day), supplemental vitamin D (0, 1–199, 200–399, 400+ IU/day), alcohol intake (0, 0.1–4.9, 5–9.9, 10+ grams/day), postmenopausal hormone use (never, past, current, unknown; for postmenopausal women only), and UV-B flux (<113, 113, >113 R-B units) (loading factors provided in Table A1). Covariate exposures reported closest to the time of blood draw were used to estimate regression coefficients. Age (years), season of blood draw (Summer, Fall, Winter, Spring), and laboratory batch were included in the regression models to account for known variation in 25(OH)D levels. Data on all components of the 25(OH)D predictor score were first available in 1986 for NHS and in 1991 for NHSII. Regression coefficients of predictors were used to calculate predicted 25(OH)D levels for each questionnaire cycle using the appropriate score based on menopausal status at each cycle. A woman did not contribute person-time to analyses over follow-up periods in which her menopausal status was unknown (total of 110,480 person-years).

### 4.4. Statistical Analysis

Women with a cancer diagnosis (except non-melanoma skin cancer), bilateral oophorectomy, or menopause due to pelvic irradiation before the baseline year (*i.e.*, 1976/1989 for the analysis of UV-B flux, 1980/1991 for the analysis of vitamin D intake, or 1986/1991 for the analysis of predicted 25(OH)D in NHS/NHSII), or unknown date of birth were excluded. Person-time of follow-up was calculated for each eligible participant from the return date of the baseline questionnaire until the date of ovarian cancer diagnosis, diagnosis of another cancer (except non-melanoma skin cancer), bilateral oophorectomy, pelvic irradiation, death, or end of follow-up (NHS: 1 June 2010; NHSII: 1 June 2011), which ever occurred first. 

To estimate long-term exposure to vitamin D, we calculated cumulative updated averages for UV-B flux, vitamin D intake, and predicted 25(OH)D to dampen variation due to measurement error [68]. Furthermore, we incorporated a 2-year time lag between exposure assessment and ovarian cancer diagnosis to reduce potential exposure changes resulting from preclinical disease. As an example to illustrate these methods, we used an average of predicted 25(OH)D levels from 1986 and 1988 to predict ovarian cancer risk from 1990 to 1992. Similarly, we averaged predicted 25(OH)D levels from 1986, 1988, and 1990 to predict risk from 1992 to 1994. As predicted 25(OH)D levels were estimated separately by menopausal status, we calculated cumulative updated average predicted 25(OH)D scores for questionnaire cycles in which a woman was premenopausal followed by a separate cumulative average variable during postmenopause. Cumulative average predicted 25(OH)D scores were then categorized into menopause-specific quintiles. Cox proportional hazards regression models, with age in months and 2-year questionnaire cycle as the time scale, were used to estimate the RR and 95% CI of ovarian cancer for each category of cumulative average UV-B flux, vitamin D intake, or predicted 25(OH)D compared to women in the lowest category of the respective exposure of interest. We assessed UV-B flux exposure at various ages (birth, age 15, age 30, study baseline) to explore whether exposure at specific times of life were more relevant to ovarian cancer risk. In secondary analyses, Cox proportional hazards competing risks models were used to investigate the associations of cumulative average exposures by major ovarian cancer histologic subtypes (serous invasive/poorly differentiated, endometrioid/clear cell, mucinous), tumor invasiveness (borderline, invasive), and tumor aggressiveness (death from invasive ovarian cancer within 3 years of diagnosis *vs*. less aggressive invasive disease). 

All multivariable models were adjusted for established ovarian cancer risk factors: number of pregnancies (continuous), duration of oral contraceptive use (never, <1 year, 1 to <5 years, 5 to <10 years, 10+ years), tubal ligation (no, yes), menopausal status (premenopausal/unknown, postmenopausal), ever use of postmenopausal hormones (no/unknown, yes), and first-degree family history of ovarian cancer (no, yes). Models assessing ovarian cancer risk associated with vitamin D intake from food were additionally adjusted for total caloric intake (continuous). We evaluated duration of estrogen-only postmenopausal hormone use (never, past use <5 years, past use 5+ years, current use <5 years, current use 5+ years), duration of breastfeeding (continuous), smoking status (never, past, current), BMI (continuous), and total physical activity (<3, 3 to <9, 9 to <18, 18 to <27, 27+ METS/week) as potential confounders. With the exception of BMI, addition of these covariates did not substantially change RR estimates and, therefore, were not included in the final models. Tests for linear trend were conducted by assigning the median exposure value to each category and treating the variable as continuous to calculate Wald’s statistic. Heterogeneity in exposure associations between cohorts was calculated using the Q statistic and DerSimonian and Laird random effects meta-analysis [69] was used to combine cohort results where appropriate. Likelihood ratio tests assessed statistical interactions by age (≤50, >50), menopausal status (premenopausal, postmenopausal), OC use status (never, ever) or duration (continuous), and BMI (<25, ≥25 kg/m^2^). We tested the proportional hazards assumption by comparing the log likelihood of models with and without interaction terms between time and the main exposures. *p*-values were based on two-sided tests and considered statistically significant at *p* < 0.05. All analyses were conducted using SAS version 9.3 (SAS Institute Inc., Cary, NC, USA). 

## 5. Conclusions

In summary, our results do not support a role for vitamin D in ovarian carcinogenesis. We observed some evidence for reduced risk of ovarian cancer associated with UV-B flux exposure among premenopausal, but not postmenopausal women. A biological rationale for a heterogenous effect of vitamin D by menopausal status is unclear, but appears consistent with observations in biomarker studies [22,49,50]. Our observation of a statistically significant increased risk of serous and less aggressive invasive tumors associated with ambient UV-B exposure in the NHS contradicts the hypothesized relationship and may be due to chance or bias as a result of an unmeasured confounder. Likewise, chance and/or bias may have contributed to the reduced risk observed among NHSII participants. Further investigation incorporating individual level UV radiation exposure data (e.g., personal sun exposure behaviors) in ovarian cancer risk within large prospective cohorts, particularly among premenopausal women, is warranted.

## Appendix

**Table A1:** Loading factors of predictors of plasma 25(OH)D levels from multiple linear regression models for premenopausal and postmenopausal women from NHS and NHSII.

	Premenopausal women		Postmenopausal women	
Covariate	N	2,431	N	3,101
R^2^		0.27		0.28
Intercept		24.32		18.53
Race/ethnicity: Asian	85	0.15	9	−1.42
Race/ethnicity: Hispanic	15	−2.59	19	−4.51
Race/ethnicity: Other	36	0.98	99	−0.09
Race/ethnicity: Black	74	−2.79	16	−10.20
Race/ethnicity: White	2,221	Ref	2,958	Ref
BMI 35+ kg/m^2^	118	−3.55	118	−5.09
BMI 30–34.9 kg/m^2^	179	−2.37	321	−2.23
BMI 25–29.9 kg/m^2^	565	Ref	935	Ref
BMI 22–24.9 kg/m^2^	753	2.22	985	1.35
BMI < 22 kg/m^2^	816	3.24	742	1.98
≥400 IU/day food vitamin D	105	4.61	197	4.02
300–399 IU/day food vitamin D	294	4.05	422	4.21
200–299 IU/day food vitamin D	699	3.05	906	3.32
100–199 IU/day food vitamin D	1,002	1.70	1,205	1.95
<100 IU/day food vitamin D	331	Ref	371	Ref
≥400 IU/day supplemental vitamin D	377	2.20	721	3.86
200–399 IU/day supplemental vitamin D	487	1.51	403	2.55
1–199 IU/day supplemental vitamin D	505	0.79	172	1.61
0 IU/day supplemental vitamin D	1,062	Ref	1,805	Ref
27+ mets/week	516	2.80	588	3.65
18 to <27 mets/week	335	1.96	437	3.37
9 to <18 mets/week	572	1.03	668	1.48
3 to <9 mets/week	592	0.16	809	1.72
<3 mets/week	416	Ref	599	Ref
Never used postmenopausal hormones		--	939	−1.60
Past postmenopausal hormone user		--	583	−1.10
Current postmenopausal hormone user		--	1,460	Ref
Unknown postmenopausal hormone use status		--	119	−0.24
UV-B flux < 113 R-B × 10^−4^	826	−0.66	1,049	−1.09
UV-B flux = 113 R-B × 10^−4^	649	−0.45	980	−1.65
UV-B flux > 113 R-B × 10^−4^	956	Ref	1,072	Ref
Alcohol: 10+ grams/day	311	2.72	574	2.09
Alcohol: 5–9.9 grams/day	299	2.26	334	0.82
Alcohol: 0.1–4.9 grams/day	1,020	1.09	1,046	0.79
Alcohol: 0 grams/day	801	Ref	1,147	Ref

## References

[1] Siegel R., Naishadham D., Jemal A. (2013). Cancer statistics, 2013. CA Cancer J. Clin..

[2] Lukanova A., Kaaks R. (2005). Endogenous hormones and ovarian cancer: Epidemiology and current hypotheses. Cancer Epidemiol. Biomarkers Prev..

[3] Fleming J.S., Beaugie C.R., Haviv I., Chenevix-Trench G., Tan O.L. (2006). Incessant ovulation, inflammation and epithelial ovarian carcinogenesis: Revisiting old hypotheses. Mol. Cell. Endocrinol..

[4] Evans R.M. (1988). The steroid and thyroid hormone receptor superfamily. Science.

[5] Deeb K.K., Trump D.L., Johnson C.S. (2007). Vitamin D signalling pathways in cancer: Potential for anticancer therapeutics. Nat. Rev. Cancer.

[6] Adams J.S., Hewison M. (2008). Unexpected actions of vitamin D: New perspectives on the regulation of innate and adaptive immunity. Nat. Clin. Pract. Endocrinol. Metab..

[7] Ahonen M.H., Zhuang Y.H., Aine R., Ylikomi T., Tuohimaa P. (2000). Androgen receptor and vitamin D receptor in human ovarian cancer: Growth stimulation and inhibition by ligands. Int. J. Cancer.

[8] Villena-Heinsen C., Meyberg R., Axt-Fliedner R., Reitnauer K., Reichrath J., Friedrich M. (2002). Immunohistochemical analysis of 1,25-dihydroxyvitamin-D3-receptors, estrogen and progesterone receptors and Ki-67 in ovarian carcinoma. Anticancer Res..

[9] Thill M., Fischer D., Kelling K., Hoellen F., Dittmer C., Hornemann A., Salehin D., Diedrich K., Friedrich M., Becker S. (2010). Expression of vitamin D receptor (VDR), cyclooxygenase-2 (COX-2) and 15-hydroxyprostaglandin dehydrogenase (15-PGDH) in benign and malignant ovarian tissue and 25-hydroxycholecalciferol (25(OH2)D3) and prostaglandin E2 (PGE2) serum level in ovarian cancer patients. J. Steroid Biochem. Mol. Biol..

[10] Schwalfenberg G.K. (2011). A review of the critical role of vitamin D in the functioning of the immune system and the clinical implications of vitamin D deficiency. Mol. Nutr. Food Res..

[11] Saunders D.E., Christensen C., Wappler N.L., Schultz J.F., Lawrence W.D., Malviya V.K., Malone J.M., Deppe G. (1993). Inhibition of c-myc in breast and ovarian carcinoma cells by 1,25-dihydroxyvitamin D3, retinoic acid and dexamethasone. Anticancer Drugs.

[12] Li P., Li C., Zhao X., Zhang X., Nicosia S.V., Bai W. (2004). p27(Kip1) stabilization and G(1) arrest by 1,25-dihydroxyvitamin D(3) in ovarian cancer cells mediated through down-regulation of cyclin E/cyclin-dependent kinase 2 and Skp1-Cullin-F-box protein/Skp2 ubiquitin ligase. J. Biol. Chem..

[13] Jiang F., Bao J., Li P., Nicosia S.V., Bai W. (2004). Induction of ovarian cancer cell apoptosis by 1,25-dihydroxyvitamin D3 through the down-regulation of telomerase. J. Biol. Chem..

[14] Lefkowitz E.S., Garland C.F. (1994). Sunlight, vitamin D, and ovarian cancer mortality rates in US women. Int. J. Epidemiol..

[15] Garland C.F., Mohr S.B., Gorham E.D., Grant W.B., Garland F.C. (2006). Role of ultraviolet B irradiance and vitamin D in prevention of ovarian cancer. Am. J. Prev. Med..

[16] Grant W.B. (2006). The likely role of vitamin D from solar ultraviolet-B irradiance in increasing cancer survival. Anticancer Res..

[17] Grant W.B. (2007). A meta-analysis of second cancers after a diagnosis of nonmelanoma skin cancer: Additional evidence that solar ultraviolet-B irradiance reduces the risk of internal cancers. J. Steroid Biochem. Mol. Biol..

[18] Grant W.B. (2010). An ecological study of cancer mortality rates in the United States with respect to solar ultraviolet-B doses, smoking, alcohol consumption and urban/rural residence. Dermatoendocrinology.

[19] Cook L.S., Neilson H.K., Lorenzetti D.L., Lee R.C. (2010). A systematic literature review of vitamin D and ovarian cancer. Am. J. Obstet. Gynecol..

[20] Merritt M.A., Cramer D.W., Vitonis A.F., Titus L.J., Terry K.L. (2013). Dairy foods and nutrients in relation to risk of ovarian cancer and major histological subtypes. Int. J. Cancer.

[21] Tran B., Jordan S.J., Lucas R., Webb P.M., Neale R. (2012). Association between ambient ultraviolet radiation and risk of epithelial ovarian cancer. Cancer Prev. Res. (Phila.).

[22] Zheng W., Danforth K.N., Tworoger S.S., Goodman M.T., Arslan A.A., Patel A.V., McCullough M.L., Weinstein S.J., Kolonel L.N., Purdue M.P. (2010). Circulating 25-hydroxyvitamin D and risk of epithelial ovarian cancer: Cohort Consortium Vitamin D Pooling Project of Rarer Cancers. Am. J. Epidemiol..

[23] Gates M.A., Rosner B.A., Hecht J.L., Tworoger S.S. (2010). Risk factors for epithelial ovarian cancer by histologic subtype. Am. J. Epidemiol..

[24] Schouten L.J., Rivera C., Hunter D.J., Spiegelman D., Adami H.O., Arslan A., Beeson W.L., van den Brandt P.A., Buring J.E., Folsom A.R. (2008). Height, body mass index, and ovarian cancer: A pooled analysis of 12 cohort studies. Cancer Epidemiol. Biomarkers Prev..

[25] Ryder N.B., Westoff C.F. (1966). Use of oral contraception in the United States, 1965. In only 5 years oral contraception has become a major means of regulating fertility. Science.

[26] Dawson D.A. (1990). Trends in use of oral contraceptives—Data from the 1987 National Health Interview Survey. Fam. Plann. Perspect..

[27] Franceschi S., La Vecchia C., Negri E., Booth M., Trichopoulos D. (1992). Ovarian cancer: Age at menopause and at first oral contraceptive use. Int. J. Cancer.

[28] Schildkraut J.M., Calingaert B., Marchbanks P.A., Moorman P.G., Rodriguez G.C. (2002). Impact of progestin and estrogen potency in oral contraceptives on ovarian cancer risk. J. Natl. Cancer Inst..

[29] Pike M.C., Pearce C.L., Peters R., Cozen W., Wan P., Wu A.H. (2004). Hormonal factors and the risk of invasive ovarian cancer: A population-based case-control study. Fertil. Steril..

[30] Lurie G., Thompson P., McDuffie K.E., Carney M.E., Terada K.Y., Goodman M.T. (2007). Association of estrogen and progestin potency of oral contraceptives with ovarian carcinoma risk. Obstet. Gynecol..

[31] Lurie G., Wilkens L.R., Thompson P.J., McDuffie K.E., Carney M.E., Terada K.Y., Goodman M.T. (2008). Combined oral contraceptive use and epithelial ovarian cancer risk: Time-related effects. Epidemiology.

[32] Tsilidis K.K., Allen N.E., Key T.J., Dossus L., Lukanova A., Bakken K., Lund E., Fournier A., Overvad K., Hansen L. (2011). Oral contraceptive use and reproductive factors and risk of ovarian cancer in the European Prospective Investigation into Cancer and Nutrition. Br. J. Cancer.

[33] Mizoue T. (2004). Ecological study of solar radiation and cancer mortality in Japan. Health Phys..

[34] Boscoe F.P., Schymura M.J. (2006). Solar ultraviolet-B exposure and cancer incidence and mortality in the United States, 1993–2002. BMC Cancer.

[35] Waltz P., Chodick G. (2008). Assessment of ecological regression in the study of colon, breast, ovary, non-Hodgkin’s lymphoma, or prostate cancer and residential UV. Eur. J. Cancer Prev..

[36] Weatherhead E.C., Tiao G.C., Reinsel G.C., Frederick J.E., DeLuisi J.J., Tam D.C.W. (1997). Analysis of long-term behavior of ultraviolet radiation measured by Robertson-Berger meters at 14 sites in the United States. J. Geophys. Res..

[37] Bodelon C., Cushing-Haugen K.L., Wicklund K.G., Doherty J.A., Rossing M.A. (2012). Sun exposure and risk of epithelial ovarian cancer. Cancer Causes Control.

[38] Lin S.W., Wheeler D.C., Park Y., Cahoon E.K., Hollenbeck A.R., Freedman D.M., Abnet C.C. (2012). Prospective study of ultraviolet radiation exposure and risk of cancer in the United States. Int. J. Cancer.

[39] Goodman M.T., Wu A.H., Tung K.H., McDuffie K., Kolonel L.N., Nomura A.M., Terada K., Wilkens L.R., Murphy S., Hankin J.H. (2002). Association of dairy products, lactose, and calcium with the risk of ovarian cancer. Am. J. Epidemiol..

[40] Kushi L.H., Mink P.J., Folsom A.R., Anderson K.E., Zheng W., Lazovich D., Sellers T.A. (1999). Prospective study of diet and ovarian cancer. Am. J. Epidemiol..

[41] Koralek D.O., Bertone-Johnson E.R., Leitzmann M.F., Sturgeon S.R., Lacey J.V., Schairer C., Schatzkin A. (2006). Relationship between calcium, lactose, vitamin D, and dairy products and ovarian cancer. Nutr. Cancer.

[42] Bidoli E., La Vecchia C., Talamini R., Negri E., Parpinel M., Conti E., Montella M., Carbone M.A., Franceschi S. (2001). Micronutrients and ovarian cancer: A case-control study in Italy. Ann. Oncol..

[43] Salazar-Martinez E., Lazcano-Ponce E.C., Gonzalez Lira-Lira G., Escudero-De los Rios P., Hernandez-Avila M. (2002). Nutritional determinants of epithelial ovarian cancer risk: A case-control study in Mexico. Oncology.

[44] Genkinger J.M., Hunter D.J., Spiegelman D., Anderson K.E., Arslan A., Beeson W.L., Buring J.E., Fraser G.E., Freudenheim J.L., Goldbohm R.A. (2006). Dairy products and ovarian cancer: A pooled analysis of 12 cohort studies. Cancer Epidemiol. Biomarkers Prev..

[45] Holick M.F. (2003). Vitamin D: A millenium perspective. J. Cell. Biochem..

[46] Bertone-Johnson E.R., Chen W.Y., Holick M.F., Hollis B.W., Colditz G.A., Willett W.C., Hankinson S.E. (2005). Plasma 25-hydroxyvitamin D and 1,25-dihydroxyvitamin D and risk of breast cancer. Cancer Epidemiol. Biomarkers Prev..

[47] Wootton A.M. (2005). Improving the measurement of 25-hydroxyvitamin D. Clin. Biochem. Rev..

[48] Wei M.Y., Giovannucci E.L. (2010). Vitamin D and multiple health outcomes in the Harvard cohorts. Mol. Nutr. Food Res..

[49] Toriola A.T., Surcel H.M., Agborsangaya C., Grankvist K., Tuohimaa P., Toniolo P., Lukanova A., Pukkala E., Lehtinen M. (2010). Serum 25-hydroxyvitamin D and the risk of ovarian cancer. Eur. J. Cancer.

[50] Toriola A.T., Surcel H.M., Calypse A., Grankvist K., Luostarinen T., Lukanova A., Pukkala E., Lehtinen M. (2010). Independent and joint effects of serum 25-hydroxyvitamin D and calcium on ovarian cancer risk: A prospective nested case-control study. Eur. J. Cancer.

[51] Scotto J., Cotton G., Urbach F., Berger D., Fears T. (1988). Biologically effective ultraviolet radiation: Surface measurements in the United States, 1974 to 1985. Science.

[52] Rich-Edwards J.W., Corsano K.A., Stampfer M.J. (1994). Test of the national death index and Equifax nationwide death search. Am. J. Epidemiol..

[53] Tworoger S.S., Hecht J.L., Giovannucci E., Hankinson S.E. (2006). Intake of folate and related nutrients in relation to risk of epithelial ovarian cancer. Am. J. Epidemiol..

[54] Scotto J., Fears T.R., Fraumeni J.F., Schottenfeld D., Fraumeni J.F. (1996). Solar radiation. Cancer Epidemiology and Prevention.

[55] Fears T.R., Bird C.C., Guerry D.T., Sagebiel R.W., Gail M.H., Elder D.E., Halpern A., Holly E.A., Hartge P., Tucker M.A. (2002). Average midrange ultraviolet radiation flux and time outdoors predict melanoma risk. Cancer Res..

[56] U.S. Department of Agriculture, Agricultural Research Service (1999). USDA nutrient database for standard reference, release 13. Nutrient Data Laboratory, Agricultural Research Service.

[57] Salvini S., Hunter D.J., Sampson L., Stampfer M.J., Colditz G.A., Rosner B., Willett W.C. (1989). Food-based validation of a dietary questionnaire: The effects of week-to-week variation in food consumption. Int. J. Epidemiol..

[58] Willett W.C., Sampson L., Browne M.L., Stampfer M.J., Rosner B., Hennekens C.H., Speizer F.E. (1988). The use of a self-administered questionnaire to assess diet four years in the past. Am. J. Epidemiol..

[59] Willett W.C., Sampson L., Stampfer M.J., Rosner B., Bain C., Witschi J., Hennekens C.H., Speizer F.E. (1985). Reproducibility and validity of a semiquantitative food frequency questionnaire. Am. J. Epidemiol..

[60] Adams J.S., Clemens T.L., Parrish J.A., Holick M.F. (1982). Vitamin-D synthesis and metabolism after ultraviolet irradiation of normal and vitamin-D-deficient subjects. N. Engl. J. Med..

[61] Feskanich D., Willett W.C., Colditz G.A. (2003). Calcium, vitamin D, milk consumption, and hip fractures: A prospective study among postmenopausal women. Am. J. Clin. Nutr..

[62] Wu T., Willett W.C., Giovannucci E. (2009). Plasma *C*-peptide is inversely associated with calcium intake in women and with plasma 25-hydroxy vitamin D in men. J. Nutr..

[63] Bertrand K.A., Giovannucci E., Liu Y., Malspeis S., Eliassen A.H., Wu K., Holmes M.D., Laden F., Feskanich D. (2012). Determinants of plasma 25-hydroxyvitamin D and development of prediction models in three US cohorts. Br. J. Nutr..

[64] Hollis B.W. (1997). Quantitation of 25-hydroxyvitamin D and 1,25-dihydroxyvitamin D by radioimmunoassay using radioiodinated tracers. Methods Enzymol..

[65] Gallicchio L., Helzlsouer K.J., Chow W.H., Freedman D.M., Hankinson S.E., Hartge P., Hartmuller V., Harvey C., Hayes R.B., Horst R.L. (2010). Circulating 25-hydroxyvitamin D and the risk of rarer cancers: Design and methods of the Cohort Consortium Vitamin D Pooling Project of Rarer Cancers. Am. J. Epidemiol..

[66] Wagner D., Hanwell H.E., Vieth R. (2009). An evaluation of automated methods for measurement of serum 25-hydroxyvitamin D. Clin. Biochem..

[67] Willett W.C., Howe G.R., Kushi L.H. (1997). Adjustment for total energy intake in epidemiologic studies. Am. J. Clin. Nutr..

[68] Hu F.B., Stampfer M.J., Rimm E., Ascherio A., Rosner B.A., Spiegelman D., Willett W.C. (1999). Dietary fat and coronary heart disease: A comparison of approaches for adjusting for total energy intake and modeling repeated dietary measurements. Am. J. Epidemiol..

[69] DerSimonian R., Laird N. (1986). Meta-analysis in clinical trials. Control. Clin. Trials.

